# A prospective observational study of optimal acupoint selection on patients with functional gastrointestinal disorders

**DOI:** 10.1097/MD.0000000000034316

**Published:** 2023-07-14

**Authors:** Heeyoung Moon, Da-Eun Yoon, Yoonjeong Seo, In-Seon Lee, Younbyoung Chae

**Affiliations:** a Department of Science in Korean Medicine, Graduate School, Kyung Hee University, Seoul, Republic of Korea; b Department of Meridian Medical Science, Graduate School, Kyung Hee University, Seoul, Republic of Korea.

**Keywords:** acupoint, functional gastrointestinal disorder, machine learning, pattern identification diagnosis, traditional Korean medicine treatment

## Abstract

Functional gastrointestinal disorders (FGIDs), which include irritable bowel syndrome (IBS) and functional dyspepsia (FD), are common gastrointestinal (GI) diseases that have a large financial impact on patients’ quality of life (QoL). Traditional Korean medicine has a particular diagnostic pattern for treating FGIDs. However, FGIDs have not been thoroughly explored because of their complexity. In this proposed study, we will investigate the acupoint selection pattern for FGID patients with various disease patterns, and further determine the best acupoints for treating FGID patients using a machine-learning algorithm.

**Methods:**

We will collect clinical data from 15 multi-center Korean medical clinics that treat FGID as part of an observational study registry. Patients who meet the criteria will be added to the registry after screening. They will receive a maximum 4-week treatment, and they will respond 3 times to a series of questions. We will investigate how doctors of FGID patients with diverse disease patterns choose the acupoints, and we will use a machine learning technique to identify the best acupoints for treating FGID patients.

**Discussion:**

This will be the first multi-center observational registry study to assess how traditional Korean medical practitioners diagnose and treat patients in the real world. The findings will shed light on how traditional Korean medicine treats FGIDs and demonstrate the rationale for the diagnostic and acupuncture treatment flow.

## 1. Introduction

At least one-third of visits to gastroenterology clinics are for functional gastrointestinal disorders (FGIDs), such as functional dyspepsia (FD) and irritable bowel syndrome (IBS).^[1]^ More than two-thirds of people have seen a doctor in the past 12 months, 40% of patients use routine medications, and one-third have undergone potentially needless abdominal surgery for their symptoms, such as a hysterectomy or cholecystectomy.^[2]^ These illnesses affect quality of life (QoL) to a degree that is comparable to that of organic gastrointestinal (GI) disease and are costly to treat.^[3]^ Many patients with FGIDs search for alternative treatments.^[4]^

Acupuncture is a non-pharmacological intervention that has been widely used to treat patients with FGIDs.^[5]^ A previous meta-analysis of FGID patients who received acupuncture treatment found that acupuncture is strongly related to improved symptoms despite the moderate or low strength of evidence.^[6]^ According to a recent meta-analysis, FD patients who received acupuncture and conventional therapy had a reduction in their symptom scores.^[7]^ Comparative Chinese trials have revealed that patients experience benefits from acupuncture compared to antispasmodic medications, despite randomized controlled trials showing no benefit of acupuncture compared to a credible sham acupuncture control for the severity or QoL of IBS symptoms.^[8]^ Patients with FD who have postprandial discomfort syndrome respond more favorably to acupuncture, particularly at certain acupoints along the stomach meridian.^[9]^ However, no prospective observational study has collected real-world data to determine the best acupoints for treating FGID patients.

The fourth version of the Rome Foundation criteria was released in 2016 and is used to diagnose and categorize FGIDs. Rather than identifying and addressing particular underlying pathophysiological mechanisms, the current methods of treating FGIDs focus on the most common GI and psychological symptoms.^[3]^ However, pattern identification is a technique used in complementary and alternative medicine sectors, such as traditional Korean medicine, to diagnose patients with FGID and reveal the precise pathophysiological pattern of the disease (diagnosis). Doctors offer patients several forms of acupuncture treatment at the outpatient department of a Korean medical clinic following the patients’ patterns.^[10]^ Significant factors have been discovered that may be used to predict the effectiveness of acupuncture for patients with FD using machine learning and support vector machine methods.^[11]^ Nonetheless, few studies have been performed on acupuncture treatment based on a pattern identification diagnosis of FGIDs.^[12]^

We have designed an observational registry study including 15 Korean medical clinics. We will investigate the pattern of acupoint selection for FGID patients with various disease patterns, and further determine the best acupoints for treating FGID patients using a machine-learning algorithm.

## 2. Methods

### 2.1. Study registration

The Clinical Research Information Service of the Korea National Institute of Health, Republic of Korea, received registration for this study (KCT0008145).

### 2.2. Study design

Up to 420 patients with a doctor-diagnosed FGID will participate in this multi-center, prospective observational registry study. This study will be conducted in 15 traditional Korean medical clinics located in the Republic of Korea. All subjects will be recruited from the daily outpatient department of each clinic participating in the study.

First, using various methods of pattern identification, we will investigate the acupoint selection patterns that doctors recommend for patients with FGIDs. Patients with FGIDs will receive a pattern identification questionnaire. Second, we will determine which acupoints are best for treating FGID. A machine learning method will be used to extract the combinations of acupoints to predict the acupuncture responders. Patients included in the study will complete a series of questionnaires a maximum of 3 times throughout their treatment period: at the first appointment (week 0), 2 weeks later (week 2), and 4 weeks later (week 4).

**Figure 1.**
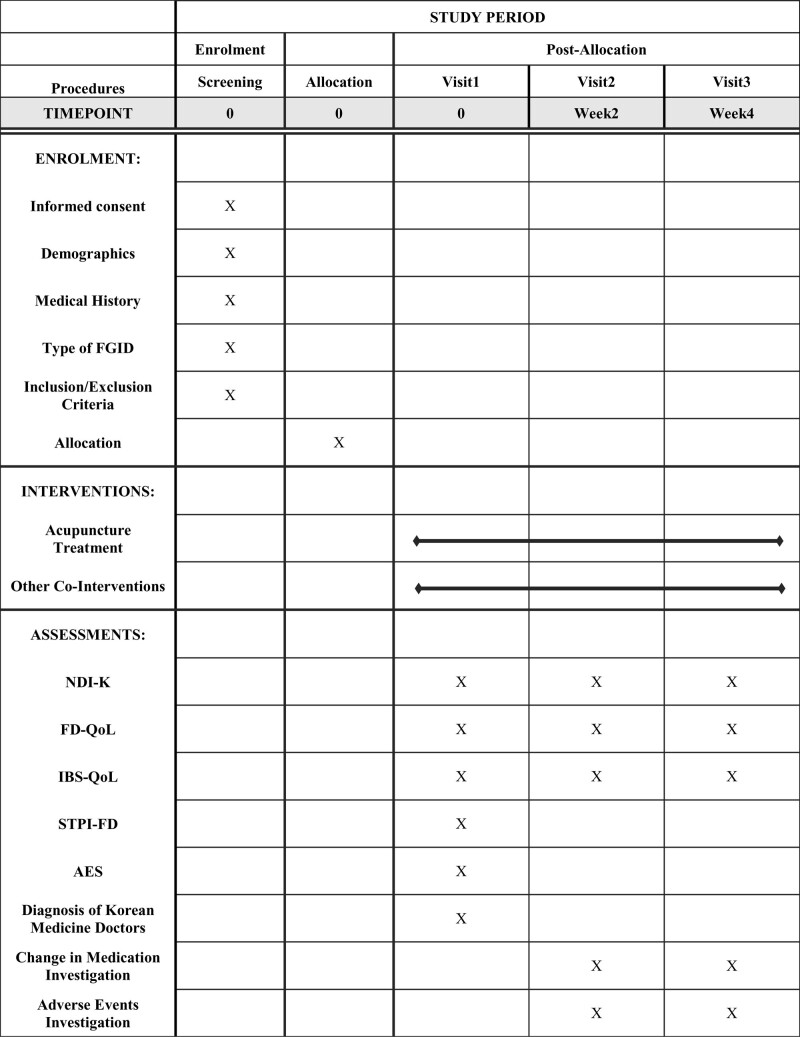
SPIRIT figure showing the schedule of enrollment, interventions and assessments. AES = acupuncture expectancy scale, FD-QoL = functional dyspepsia quality of life, IBS-QoL = irritable bowel syndrome quality of life, NPI = Nepean Dyspepsia Index, STPI-FD = standard tool for pattern identification of functional dyspepsia.

After receiving an explanation of the advantages and disadvantages of participating in the study, the patients will be requested to sign an informed consent form in writing during their initial appointment. They will be assessed for compliance with the inclusion and exclusion criteria in a screening procedure. Eligible subjects will be added to the registry after screening. The patients will receive combined Korean medical treatment for a maximum of 4 weeks, which will include acupuncture treatment and the responses to a series of questionnaires. The detailed study procedure is shown in Figure 1.

### 2.3. Participants

Adults aged 18 to 70 years will be the subjects of this registry study. Following a thorough description of the study purpose and procedures, participants will sign a written consent form.

#### 2.3.1. Inclusion criteria.

Patients diagnosed with FD (either epigastric pain syndrome or postprandial distress syndrome) or IBS via the Rome Ⅳ Criteria, who can consistently visit a clinic for treatment, and who voluntarily agree to participate will be included in the study.

#### 2.3.2. Exclusion criteria.

The following patients will be excluded: those with an organic disease that induces dysfunction of the GI tract (e.g., central nervous system disease, neoplastic disease, metabolic disease, inflammatory disease of the GI tract, or taking medicine that affects GI tract function), who have received active treatment (e.g., hospitalization for severe FGID symptoms) within 3 months before visiting the outpatient Korean medical clinic, and who have participated in other studies. In addition, patients who are considered inappropriate to participate, based on the decision of the doctor, will also be excluded.

#### 2.3.3. Recruitment of the study participants.

All adult patients seeking treatment for FGID at the outpatient division of any of the 15 participating Korean medical clinics will be eligible to join the patient group. Each clinic will receive a supply of posters with advertisements. These posters will be used to inform visiting patients.

#### 2.3.4. Registration of participants.

FGID patients who match the requirements will be added to the registry. The medical data of the participants related to FGID will be collected during their medical care after they have enrolled in the registry.

#### 2.3.5. Pattern identification types of patients.

Patients will be categorized into 6 pattern identification types, as per the clinical practice guideline of Korean medicine for FD: spleen and stomach deficiency and cold pattern; spleen deficiency with qi stagnation pattern; liver-stomach disharmony pattern; tangled cold and heat pattern; pattern of dampness and heat in the spleen and stomach systems; and food retention pattern. The Standard Tool for Pattern Identification of Functional Dyspepsia (STPI-FD) questionnaire will be employed to determine the patient pattern.^[13]^ This questionnaire consists of 12 categories of questions reflecting various symptoms of FGIDs. This questionnaire will be presented to patients only once at the first visit (0 weeks).

### 2.4. Intervention

Acupuncture will be used to treat all FGID patients as determined by the doctor. Patients may receive extra treatment depending on the doctor decision.

#### 2.4.1. Acupuncture treatment.

Based on the top 10 frequently used acupoints according to previous systematic reviews, examples of acupoints (e.g., CV12, ST25, ST36, CV10, LR3, BL20, PC6, GV7, BL21, and SP6) will be shown in the case report form. Pattern identification will be used to choose the acupoints, and more acupoints will be permitted based on the patient additional symptoms. There will be no restrictions on the number of acupoints used for treatment. Korean medical experts with at least 5 years of clinical practice will deliver all acupuncture treatments.

#### 2.4.2. Other co-interventions.

Herbal acupuncture, herbal medicine, cupping therapy, moxibustion, and other treatments will also be provided simultaneously in clinics in addition to traditional acupuncture. Co-interventional data will be considered a covariate when estimating the impact of the treatment.

### 2.5. Outcomes

The Rome IV criteria will be used first to diagnose FGID. Patients who are assigned to this registry will be required to complete 3 evaluations of the Korean versions of the Nepean Dyspepsia Index,^[14]^ FD-QoL,^[15]^ and IBS-QoL questionnaires.^[16]^ The Nepean dyspepsia index questionnaire includes 15 questions on different dyspepsia symptoms that assess their frequency, seriousness, and subjective perception. Total of 21 questions divided into four areas, including nutrition, vitality, emotions, and social function, in FD-QoL questionnaire reflect how the QoL of FD patients has changed. In all, 34 items on how the QoL of IBS patients has changed are included in the IBS-QoL questionnaire. In addition, the acupuncture expectancy scale will be evaluated at baseline to gauge the general effects of treatment.^[17]^

### 2.6. Sample size and data analysis

Although sample size calculations are not necessary for observational registry studies, we have determined that 420 individuals are needed, considering the volume of business at the outpatient clinics taking part in this study.

Each outcome measure will be compared to the clinical outcome between the baseline and termination, and the clinical outcome will be a dependent variable. Combinations of the applied acupoints will act as independent factors to predict the most effective acupoints for the acupuncture treatment. To improve the effectiveness of acupuncture treatments for FGID patients, the properties of the acupoints will be extracted using machine learning algorithms, such as support vector machine and logistic regression models.

### 2.7. Data collection and monitoring

All data will be gathered in case report format on printed paper and will be managed in a secure online database. The Mytrial system (http://www.mytrial.co.kr, Bethesdasoft Co., Seoul, Korea) will be used to manage the electronic data. With a user identification and password, only authorized personnel are able to access the database. The data management system will enable automatic editing to record and audit electronic data. The monitoring committee, which does not have any competing interests, will periodically review all of the study documents, including informed consent forms and case report forms containing the data collected.

All study materials will be identified by special codes rather than by participant names to maintain anonymity. Following the release of the results, all data containing participant information will be securely stored for 3 years.

### 2.8. Ethical issues

The institutional review board committee at Kyung Hee University in Seoul, Republic of Korea, has approved this study (KHSIRB-22-074RA). The Declaration of Helsinki will be followed while conducting the study. After receiving a thorough explanation of the study goals, participants’ rights, potential adverse events (AEs), and safety protection, participants will receive consent forms. All participants will have to sign the documents to be enrolled. A separate compensation award will be established in the event of an emergency or serious AE, and patients will receive the best care at each clinic. Additionally, participants will have the option to cancel their participation at any time.

### 2.9. Adverse events

Any mild or significant AE connected with the study will be recorded, along with their frequency and seriousness. After receiving acupuncture therapy, patients will be informed of any potential AEs, such as discomfort, bruising, and bleeding. The principal investigator will evaluate any associations between the AEs and the trial.

### 2.10. Changes to the study protocol

The institutional review board committee will approve any modifications to the protocol before they can be implemented because they may impact the effectiveness of the study, its potential benefits, or the safety of the subjects. Modifications could include changes to the study design, sample size, or procedures.

## 3. Discussion

This study will collect clinical data from 15 multicenter Korean medical clinics that treat FGID as part of an observational study registry. With a machine learning system, we will further determine the best acupoints for treating FGID patients after analyzing the acupoint selection patterns.

Depending on the pattern, multiple acupuncture treatments for the same disease may be suggested by traditional Korean medicine. In a previous study, 69 Korean clinicians were asked to provide diagnoses for hypothetical patients based on the clinical information from a previous study that employed a traditional Korean medicine diagnostic algorithm.^[10]^ The most often used acupoints for individuals with FD were CV12, LI4, LR3, ST36, and PC6 but the choices varied depending on which of the 3 patterns (liver qi depression, spleen-stomach weakness, and food accumulation/phlegm-fluid retention) was present. Ha^[13]^ created a 36-item test for studying functional dyspepsia (STPI-FD) based on the Delphi technique and the opinions of specialists, and from which 6 types of FD were distinguished: spleen and stomach deficiency and cold, spleen deficiency with qi stagnation, liver-stomach disharmony, tangled cold and heat pattern, dampness and heat in the spleen and stomach, and food retention pattern. Depending on the pattern identified, supervised learning will be used to extract important clinical features. For our proposed study, we anticipate that the data collected from the 15 different clinics will reveal various FGID patient patterns and further illustrate the patterns of the chosen acupoint combinations. To identify the optimum acupoints for disorders such as FGID, pattern identification based on supervised learning will be used to extract crucial information from real-world clinical data.

Next, the best acupoints for treating FGID with acupuncture will be predicted using machine learning and the patient results. Individuals respond to acupuncture in different ways.^[18]^ As mentioned above, machine learning can be used to forecast the best acupoints based on patient outcomes. In a previous study, the effectiveness of acupuncture was predicted using a support vector machine for individuals with FD.^[11]^ Gender, disease subtype, and education level were significant predictors of treatment results. Similarly, machine learning will be used to identify those who are more likely to respond to acupuncture, or those who will improve (display minimally clinically significant differences) from baseline following 4 weeks of acupuncture treatment. Data from 80% of the patients with FD will be used to create a predictive model, and the data from the other 20% will be used to validate the model and assess its performance. The performance of supervised machine learning models, such as support vector machine and logistic regression models, will be assessed using accuracy, sensitivity, and specificity analyses. Machine learning can be used to predict the optimal acupoints for each patient and help doctors assess treatment responses.

## Author contributions

**Conceptualization:** Heeyoung Moon, Yoonjeong Seo, Younbyoung Chae.

**Investigation:** Heeyoung Moon, Da-Eun Yoon.

**Supervision:** In-Seon Lee, Younbyoung Chae.

**Writing – original draft:** Heeyoung Moon, Younbyoung Chae.

**Writing – review & editing:** Da-Eun Yoon, In-Seon Lee.
